# Preventive Treatment with Astaxanthin Microencapsulated with Spirulina Powder, Administered in a Dose Range Equivalent to Human Consumption, Prevents LPS-Induced Cognitive Impairment in Rats

**DOI:** 10.3390/nu15132854

**Published:** 2023-06-23

**Authors:** Miquel Martin, Matteo M. Pusceddu, Joan Teichenné, Teresa Negra, Alan Connolly, Xavier Escoté, Helena Torrell Galceran, Adrià Cereto Massagué, Iris Samarra Mestre, Antoni del Pino Rius, Jordi Romero-Gimenez, Cristina Egea, Juan Maria Alcaide-Hidalgo, Josep Maria del Bas

**Affiliations:** 1 Eurecat—Centre Tecnològic de Catalunya, Unitat de Nutrició i Salut, 43204 Reus, Spain; miquel.martin@eurecat.org (M.M.); matteo.pusceddu@gmail.com (M.M.P.); joan.teichenne@eurecat.org (J.T.); xavier.escote@eurecat.org (X.E.); jordi.romerog@eurecat.org (J.R.-G.); cristina.egea@eurecat.org (C.E.); juanmaria.alcaide@eurecat.org (J.M.A.-H.); 2 Lubrizol Nutraceuticals, 08850 Gavà, Spain; teresa.negra@lubrizol.com (T.N.); alan.connolly@lubrizol.com (A.C.); 3 Eurecat—Centre Tecnològic de Catalunya, Centre for Omic Sciences, Joint Unit Eurecat—Universitat Rovira i Virgili, Unique Scientific and Technical Infrastructure (ICTS), 43204 Reus, Spain; helena.torrell@eurecat.org (H.T.G.); adria.cereto@ce.eurecat.org (A.C.M.); iris.samarra@eurecat.org (I.S.M.); antoni.delpino@eurecat.org (A.d.P.R.); 4 Eurecat—Centre Tecnològic de Catalunya, Biotechnology Area, 43204 Reus, Spain

**Keywords:** astaxanthin, microencapsulation, spirulina, cognitive impairment, Morris Water Maze, microglia, neuroinflammation, LPS, microbiota

## Abstract

Cognitive alterations are a common feature associated with many neurodegenerative diseases and are considered a major health concern worldwide. Cognitive alterations are triggered by microglia activation and oxidative/inflammatory processes in specific areas of the central nervous system. Consumption of bioactive compounds with antioxidative and anti-inflammatory effects, such as astaxanthin and spirulina, can help in preventing the development of these pathologies. In this study, we have investigated the potential beneficial neuroprotective effects of a low dose of astaxanthin (ASX) microencapsulated within spirulina (ASXSP) in female rats to prevent the cognitive deficits associated with the administration of LPS. Alterations in memory processing were evaluated in the Y-Maze and Morris Water Maze (MWM) paradigms. Changes in microglia activation and in gut microbiota content were also investigated. Our results demonstrate that LPS modified long-term memory in the MWM and increased microglia activation in the hippocampus and prefrontal cortex. Preventive treatment with ASXSP ameliorated LPS-cognitive alterations and microglia activation in both brain regions. Moreover, ASXSP was able to partially revert LPS-induced gut dysbiosis. Our results demonstrate the neuroprotective benefits of ASX when microencapsulated with spirulina acting through different mechanisms, including antioxidant, anti-inflammatory and, probably, prebiotic actions.

## 1. Introduction

In the last decades, human life expectancy has experienced a significant increase in most developed countries, because of better health care and hygiene, healthier lifestyles, and proper access to food, among other factors [1]. However, the frequency and prevalence of many neurodegenerative diseases (ND) associated with aging, such as Alzheimer’s disease, has also increased [2]. In fact, aging is considered a major risk factor for the onset and progression of many of these pathologies [3]. Nowadays, the lack of an efficient treatment for most ND represents a major health concern, making the search for new approaches to ameliorate or prevent their development a necessity [4].

Neurodegenerative diseases are a heterogeneous group of disorders that share, as a common hallmark, important alterations in cognition. These alterations are due to different mechanisms, including the aberrant activation of microglia cells and the subsequent increase in oxidative and inflammatory processes in areas of the central nervous system (CNS) involved in learning and memory, such as the hippocampus and prefrontal cortex (mPFC) [5,6]. 

Microglial cells, the resident macrophages of the CNS, are key components of the CNS immune system. In normal physiological conditions, activation of the immune system occurs as a protective reaction against microbial infections, acute injury, or disease. However, uncontrolled, and sustained microglia activation can occur during the development of different pathologies, including ND, with important deleterious consequences. Thereby, sustained microglia activation increases synapse pruning, produces inflammation, and creates a toxic environment that threatens correct neuronal functionality leading to impairments in the learning and memory processes [7,8]. In fact, aberrant changes in microglia function are considered key pathological hallmarks of ND [9]. 

In recent decades growing evidence points to the role of the gut microbiome as a potential novel modulator of CNS activity by mechanisms that include the synthesis and release of different neuroactive compounds, changes in the permeability of the blood brain barrier (BBB), the inflammatory status of the CNS, the contribution to the maturation and activation of host microglia cells, among others [10]. Perturbations in the normal gut microbiota balance (gut dysbiosis) can contribute to the pathophysiology of mental illnesses, including ND, and favoring the presence of beneficial microbiota through the consumption of prebiotics has been proven to exert positive benefits on cognition [10]. 

Healthy eating habits can help maintain a positive health status, improve brain functionality, and prevent the development of cognitive decline associated with ND. In this sense, the Mediterranean and MIND (Mediterranean-DASH Diet Intervention for Neurodegenerative Delay) diets, which are characterized by the high consumption of fruits and vegetables, olive oil, and the low intake of saturated fat and cholesterol, among others, are associated with better cognitive and memory performance, and a lower risk of ND. It is suggested that these effects rely on the important anti-inflammatory effects of these diets, due to their high content of antioxidants and anti-inflammatory nutrients [11]. Different dietary nutrients have been proven to exert neuroprotective effects by modulating the oxidative/inflammatory state of the CNS [12,13], or by potentiating the number of beneficial gut microbiota demonstrating a prebiotic effect [14]. These factors combined make them an interesting strategy to prevent cognitive deterioration. Carotenoids, such as astaxanthin (ASX), are among these family members. ASX is present in large quantities in microalgae, such as *Haematococcus pluvialis*, but is also present in marine animals such as salmonids, shrimp, and crayfish. This carotenoid exerts anti-inflammatory actions by inhibiting the release of different interleukins (ILs), tumor necrosis factor alpha (TNFα), as well as by potentiating the release of different antioxidant elements, including the Nrf2/antioxidant response elements (Nrf2/ARE) [15,16]. Moreover, previous studies have suggested that ASX can also exert beneficial neurobiological effects as a potential prebiotic. In fact, recent investigations have reported modifications in gut dysbiosis after exposure to ASX in obese mice fed with a high-fat diet [17]. Clinical studies have also provided evidence on the potential beneficial effects due to the consumption of this carotenoid. These studies highlight ASX as a promising dietary supplement with excellent safety and tolerability that can reduce oxidative stress in overweight and obese subjects, and in smokers. ASX has been shown to lower different inflammatory biomarkers, while boosting the immune response in the tuberculin skin test, while also improving cholesterol and blood microcirculation [18,19,20,21]. ASX has also been shown to improve cognition in small clinical trials in patients with mild cognitive impairment [22]. This emphasizes the need to supplement diets with natural ASX rich sources, such as those found in dietary supplements. However, ASX antioxidant and anti-inflammatory properties are highly sensitive to external factors that can accelerate its degradation and isomerization. Therefore, to protect and stabilize the beneficial properties of ASX, a microencapsulation step is recommended [23]. Microencapsulation involves the entrapment of an active ingredient within a wall matrix, which can provide protection or functionality for the active ingredient. Spirulina (*Spirulina platensis*), a microalga with important nutritional properties, is a good candidate as an encapsulating matrix. Microencapsulation of ASX in a matrix of spirulina provides a beneficial environment to protect the sensitive carotenoid, while possibly also resulting in a synergistic bioactive effect. This microalga contains essential amino acids, phycocyanin, vitamins, carotenoids, and minerals with important anti-inflammatory, antioxidant, and neuroprotective properties [24]. Like ASX, the consumption of spirulina can also be beneficial as a prebiotic on human gut microbiota. In fact, recent studies have demonstrated that chronic oral administration of spirulina can modify the diversity of gut microbiota, favoring the presence of beneficial bacteria families and maintaining microbial homeostasis [25]. 

The aim of this study was to characterize the neuroprotective effects of ASX microencapsulated within a spirulina matrix (ASXSP, commercially referred to as ASTAGILE™ microcapsules) in rats exposed to an animal model of neuroinflammation-induced cognitive impairment after LPS administration. This is one of the best characterized animal models to investigate the deleterious effects of neuroinflammatory processes on cognition in rodents [26]. LPS is a component of the outer membrane of gram-negative bacteria that induces neuroinflammation via the microglial TLR-4 signaling pathway, increasing the release of proinflammatory cytokines (such as IL-1, IL-6, IL-12, and TNF-α), and chemokines (CCL2, CCL5, and CXCL8), among others. As a final effect, glutamatergic neurotransmission is jeopardized, a mechanism suggested to produce the learning and memory deficits associated with exposure to LPS [27,28]. Chronic administration of LPS produces cognitive dysfunction in rats when evaluated on associative and spatial learning tasks [29,30]. In the present study, the rats received a preventive schedule of treatment with a low dose of ASXSP, at an equivalent dosage of ASX to that reported to produce beneficial effects in human subjects. Our results demonstrate that this low dose of ASX in combination with spirulina was able to ameliorate LPS-induced cognitive dysfunction in rats and demonstrates the important neuroprotective effects from the combination of this carotenoid and microalga, emphasizing its relevance as a basic nutrient. 

## 2. Materials and Methods

### 2.1. Animals

Forty female (7-week-old) Sprague Dawley rats (Envigo RMS Spain, Barcelona, Spain) were maintained in the local animal unit. Food and water were available ad libitum, and the rats were maintained on a normal 12:12 h light/dark cycle with the temperature at 22 ± 1 °C. The rats were single housed in plastic cages with sawdust bedding in an enriched environment, with shredded paper and a cardboard roll. The rats were left undisturbed for one week, except for routine cage cleaning, twice per week, and weekly body weight measurement, until treatments. After the experiments, the adult rats were fasted for 5 h, (from 09.00 to 14.00 h), without restriction on water and killed by decapitation.

### 2.2. Protocol

One week after acclimation to the local animal unit, the animals were randomly allocated to the following treatments: control vehicle (water, p.o., *n* = 20) or ASXSP (ASTAGILE™ microcapsules 80 mg/kg/day (with a total ASX intake of 1.2 mg/kg/day and 66 mg/kg/day of spirulina), p.o., *n* = 20). ASX was obtained from the *Haematococcus pluvialis* microalga and encapsulated in a matrix of organic *Arthrospira platensis* (Spirulina) powder to increase stability and maintain its antioxidant properties. Moreover, this microencapsulation increased, by synergic mechanisms, the antioxidant capacity of ASX due to the presence of water-soluble antioxidants in spirulina, such as phycocyanin. ASXSP was delivered considering a low dose of ASX of 1.2 mg/kg/day for 42 days. This dose of ASX was selected according to clinical evidence, where a maximum of 12 mg/day was shown to exert positive effects on different cognitive tasks in human patients [31,32,33]. By applying the Reagan-Shaw formula [34], we then extracted a dose of 1.2 mg/Kg of body weight in rats from the human dose of 12 mg/day. As commented above, the rats also ingested spirulina at a dose of 66 mg/kg/day. The ASXSP treatments were dissolved in water and administered by oral route, using a micropipette, until the end of the study. The controls were administered with tap water.

Three weeks after the beginning of the treatments, half of the cohort for each experimental group (*n* = 10) underwent LPS treatment (500 µg/kg, Sigma-Aldrich: ref. # L2630 (St. Louis, MI, USA)) by intraperitoneal injection, for seven days. The other half of the animals in each cohort were injected intraperitoneally with saline (0.9%) for the same time period. 

**Scheme 1:**
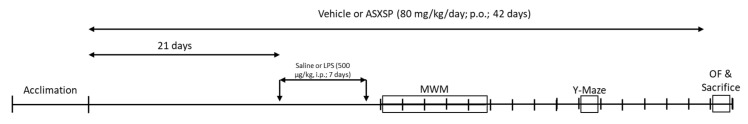
Scheme describing the experimental protocol used in this study.

All the treatments were administered every day, between 09.00 and 10.00 h. The animals underwent behavioral testing the day after the last LPS administration, beginning with exposure to the Morris Water Maze (MWM) paradigm. Ten days after the last LPS administration, the rats were exposed to the Y-Maze test. Six days after the Y-Maze test, the animals were exposed to the Open Field (OF) and, immediately after, sacrificed. The ASXSP or control vehicle (water) treatments lasted until the day of the sacrifice (see Scheme 1). 

### 2.3. Behavioral Evaluations

#### 2.3.1. Morris Water Maze Test

The rats were exposed to the MWM one day after the last administration of LPS and underwent the training protocol for five days. This test assesses the spatial learning and references memory in rodents across repeated training trials, which relies on distal visual clues to navigate from start locations around the perimeter of an open swimming arena to locate a submerged escape platform. 

The test was carried out using a protocol like others previously published [35]. The apparatus consisted of a circular tank 150 cm in diameter × 58 cm high, filled up with water (23–25 °C, 40 cm deep). A transparent platform with a diameter of 10 cm was placed in the middle of one of the quadrants, slightly submerged below the water level. The water was colored white with a non-toxic white paint to hide the platform. Distal cues were arranged around the maze to provide landmarks that the animals could use to navigate to the platform. The animals received 5 days of training that consisted of 4 trials/day. At the beginning of each trial, the animal was placed in one of four start positions facing the wall of the tank and allowed to explore the maze for 60 s. A different starting position was used for each of the four trials on a given day, arranged in a semi-random pattern. Once the rats reached the platform, they were held there for 20 s. If the platform was not found within the given time, the rats were gently assisted to the platform by the experimenter and detained there for 20 s. The amount of time needed to reach the platform was video recorded and analyzed using a tracking system (ANY-Maze, version 4.82). Data from the scape latency throughout the 5-day training period was also calculated as the area under the curve (AUC). The AUC was calculated by using a standard trapezoidal method. The following equation was used: AUC = [0.5 × (B1 + B2) × h] + [0.5 × (B2 + B3) × h] +…+ [0.5 × (Bn + Bn + 1) × h], where Bn were the infusions received for each rat and h was the time (days) that passed between the consecutive measurements [36].

#### 2.3.2. Y-Maze Test

This test is based on the natural drive of rodents to explore novel environments and was used to evaluate spatial reference memory [37]. The rats were exposed to this paradigm 10 days after the last LPS administration. The maze consisted of three identical arms mounted in a Y shape. During the 5 min habituation phase, the rats were placed at the end of one of the arms (“start arm”) facing the wall. The rats were allowed to explore two arms of the Y-maze, while the entry into the third arm was blocked. After the habituation phase, the rats were returned to their home cage for a 1 h inter-trial interval. During the test phase, the rats were allowed to explore all 3 arms of the maze (including the novel 3rd arm, previously blocked). The time and number of entries into the novel arm was analyzed using a tracking system (ANY-Maze, version 4.82). A rat with no preference for any of the arms during the testing session is an indication of an impaired spatial memory, which may indicate impaired functioning of the hippocampus.

### 2.4. Sacrifice, Tissue Extraction, and Processing

The rats were fasted for 5 h (with ad libitum access to water) and sacrificed by decapitation after exposure to the Open Field test. Immediately after decapitation, the brain was removed and paraffin embedded to perform immunohistochemical staining of the microglial cells in both the hippocampus and mPFC. The hippocampal and cortical coronal sections were defined in accordance with Paxinos’ rat brain atlas and cut (5 μm) by a sliding microtome. The hippocampal and cortical sections were stained with a specific antibody against microglial cells (CD68, Bio SB Clona BSB-8, catalog # BSB 5290 (Goleta, CA, USA)). Ten high-magnification field (400×) images were taken per slide (approximately 2 mm^2^). The relative integrated density (RID) was analyzed per section, as a semiquantitative evaluation of microglia activation. 

In addition, cecum samples were isolated and immediately frozen until further processing. To perform a gut microbiota analysis, DNA was extracted from the cecum stool samples using a QIAamp DNA Stool Mini Kit (Qiagen Inc., Hilden, Germany), according to the manufacturer’s instructions. The DNA purity and integrity were assessed using spectrophotometry (NanoDrop, Thermo Fisher Scientific, Waltham, MA, USA). The DNA samples were used for 16S rRNA gene amplification and purification. Two variable regions (V3, V4) of the 16S rRNA gene were PCR amplified using primer pair 341F-532R (5′-CCTACGGGRSGCAGCAG-3′, 5′-ATTACCGCGGCTGCT-3′), which targets the V3 region and primer pair 515F-806R (5′-GTGCCAGCMGCCGCGGTAA-3′, 5′-GGACTACHVGGGTWTCTAAT-3′), which targets the V4 region. The coordinates were based on the 16SrRNA gene of the *Escherichia coli* strain K-12 substr. MG1655. These primers needed to be redesigned to include each adaptor sequence at 5′ end used in the Ion Torrent sequencing library preparation protocol and a barcode sequence of ten bases for samples differentiation. Each 12.5 μL PCR reaction mixture consisted of genomic DNA (50 ng), 6.75 μL AmpliTaq Gold 360 Master Mix (Applied Biosystems, Thermo Fisher Scientific, Waltham, MA, USA), and 0.75 μL of each primer (5 μM). PCR was performed in a Veriti Thermal Cycler (Applied Biosystems, Thermo Fisher Scientific, Waltham, MA, USA) with the following cycling conditions: 5 min at 95 °C, 30 cycles of 30 s at 95 °C, 30 s at 55 °C, and 90 s at 72 °C, ending with 2 min at 72 °C. PCR products were analyzed using 2% agarose gel electrophoresis and further DNA band purification was performed with NucleoSpin (Macherey-Nagel, Berlin, Germany). The Agilent TapeStation (Agilent Technologies, Santa Clara, CA, USA) and the associated Agilent DNA 1000 ScreenTape High Sensitivity and High Sensitivity DNA Reagents (Agilent Technologies, Santa Clara, CA, USA) were used to determine the quality, length, and concentration of the libraries needed for the sequencing procedure. Once the individual libraries were created, they were mixed in equimolar amounts.

### 2.5. Statistical Analysis

All the data are expressed as mean ± standard error of the mean (SEM). A two-way ANOVA of repeated measure (RM)-ANOVA, two-way ANOVA, and one-way ANOVA were used to analyze the results obtained in the MWM, Y-Maze, Open Field, and immunohistochemical experiments. The level of statistical significance was set at bilateral 5% (*p* < 0.05). All the statistical analyses were performed with the SPSS Statistics version 22 software (SPSS Inc., Chicago, IL, USA).

Ion Torrent and data analysis for gut microbiota sequencing analysis. The multiplexed mixture of twenty samples were diluted to 50 pM DNA concentration prior to clonal amplification. The Ion 520 & Ion 530 Kit-Chef (Life Technologies, Carlsbad, CA, USA) was employed for template preparation and sequencing, according to the manufacturer’s instructions. Each mixture of prepared samples was loaded onto a 530 chip and sequenced using the Ion S5 system (Life Technologies, Carlsbad, California, USA). After sequencing, the Ion Torrent Suite software removed the low quality and polyclonal sequences and those reads were then analyzed using QIIME, the analysis included OTUs (operational taxonomic units) clustering, alpha-diversity analysis, OTUs analysis and species annotation, and beta-diversity analysis. For each taxonomic level, the relative abundances between the groups were compared with the Kruskal–Wallis test.

## 3. Results

### 3.1. Preventive Treatment with ASXSP Ameliorates Long-Term, but Not Short-Term Memory Alterations Associated with the Exposure to LPS

**Figure 1:**
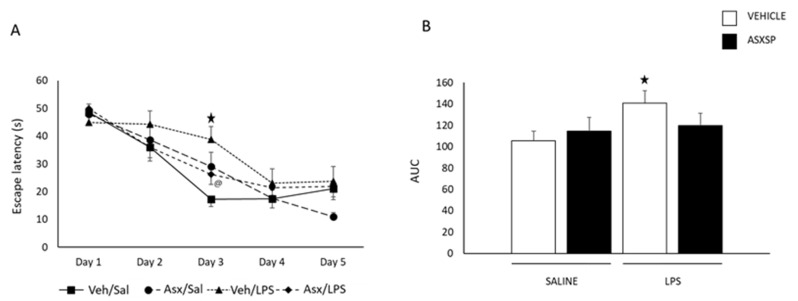
Chronic treatment with ASXSP ameliorates LPS-induced cognitive impairment in the MWM paradigm. (**A**) Scape latency on the 5 training days in the MWM for the four experimental groups: vehicle saline (Veh/Sal), ASXSP saline (Asx/Sal), vehicle LPS (Veh/LPS), and ASXSP LPS (Asx/LPS) rats. (**B**) Area under the curve (AUC) of the latency to find the hidden platform on the 5 training days in the MWM. Data are presented as the mean ± SEM (*n* = 10 animals/group). ★, *p* < 0.05 vs. vehicle saline group. @, *p* < 0.05 vs. vehicle LPS group (one-way ANOVA).

Long-term memory was evaluated in the MWM paradigm. The results from our study demonstrate that training for 5 days resulted in an improvement in the learning process in the control rats (vehicle-saline group), which showed the lowest escape latency to reach the platform on the third training day (Figure 1A). In contrast, chronic LPS treatment produced an important cognitive dysfunction in the rats, an effect that was especially relevant also on third training day, where the difference with the control vehicle saline rats was higher (Figure 1A). Interestingly, preventive treatment with ASXSP ameliorated the LPS-cognitive alterations, with no major effects in the control vehicle rats. When statistical analysis was performed, the two-way ANOVA of repeated measures showed an effect from the day (F_(4,32)_ = 64.336, *p* < 0.001), with no effect from the exposure to LPS (F_(1,35)_ = 2.890, n.s.) or the exposure to ASXSP (F_(1,35)_ = 0.340, n.s.), and a significative interaction between the factors (F_(1,35)_ =518.385, *p* < 0.001). These results indicate that the observed differences are due to the exposure to the treatment over time. When the responses between the experimental groups were separately analyzed on each training day, the two-way ANOVA revealed a significant difference on day 3 (Figure 1A, two-way ANOVA, F_(3,35)_ = 4463, *p* < 0.05)). The posterior one-way ANOVA revealed a significant difference between the control vehicle saline and vehicle LPS rats (F_(1,17)_ = 15.512, *p* < 0.05). Moreover, pretreatment with ASXSP improved the performance by LPS exposed animals when compared to the vehicle LPS rats on the same day (F_(1,18)_ = 4.668, *p* < 0.05).

The area under the curve (AUC) for the entire period of training (5 days) was also evaluated for each experimental group (Figure 1B). The two-way ANOVA revealed no changes between the experimental groups (F_(3,35)_ =1.656, n.s.). However, a significant difference was observed in the control vehicle saline group when compared to the vehicle LPS rats during the one-way ANOVA analysis (F_(1,17)_ = 5.507, *p* < 0.05). This difference was partially reverted by the preventive treatment with ASXSP, as no difference with the control vehicle saline rats was revealed.

**Figure 2:**
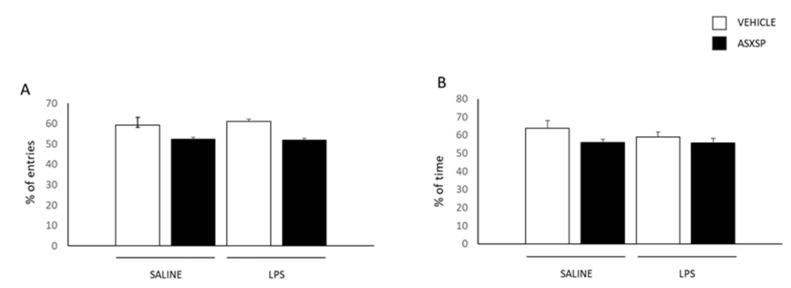
Exposure to LPS or ASXSP alone, or in combination, produced no effects on short-term recognition memory in the Y-maze. (**A**) Number of entries in the novel arm expressed as the exploration ratio. (**B**) Time spent in the novel arm expressed as the exploration ratio. Data are expressed as mean ± SEM (*n* = 10 animals/group).

In addition, the rats were exposed to the Y-maze to evaluate their short-term spatial reference memory and the effects of the exposure to LPS and ASXSP. No differences were observed between the experimental groups in the % of entries and time in the new arm (Figure 2A,B). These results suggest that exposure to LPS or ASXSP alone, or in combination, produce no alterations in short-term memory processes.

Overall, our data suggest that exposure to LPS can produce important dysfunctional effects on long-term, but not short-term, memory processing. Moreover, pre-treatment with ASXSP can revert the deleterious effect from the exposure to LPS.

### 3.2. ASXSP Modulates LPS-Induced Microglial Cell Activation in the Hippocampus and mPFC

**Figure 3:**
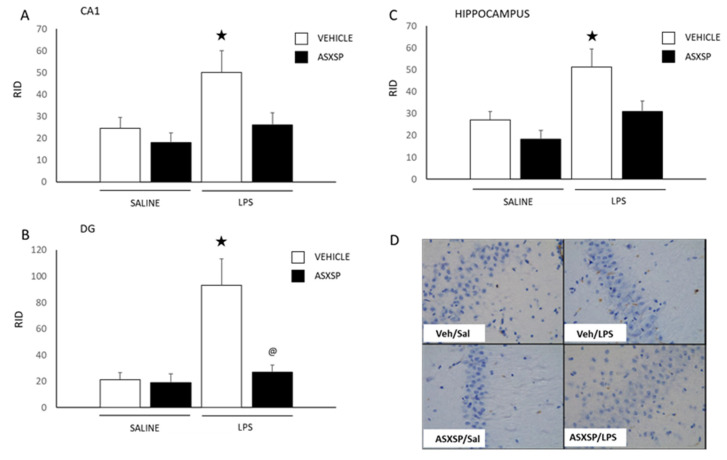
Preventive treatment with ASXSP ameliorates LPS-induced microglia activation in the hippocampus. The relative integrated density (RID) of the CD68 positive cells in (**A**) the CA1 region, (**B**) the DG region, and (**C**) the whole hippocampus. (**D**) The representative photomicrographs of CD68 staining in the hippocampus in the different experimental groups. Data are expressed as mean ± SEM (*n* = 10 animals/group). ★, *p* < 0.05 vs. vehicle saline group. @, *p* < 0.05 vehicle LPS group (one-way ANOVA).

**Figure 4:**
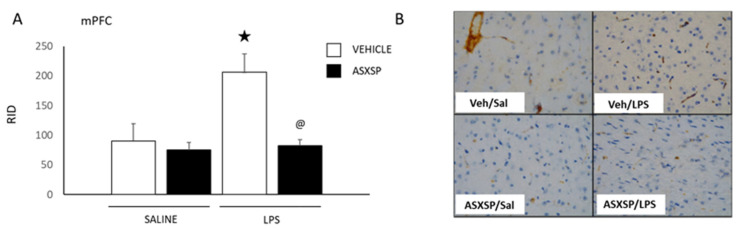
Preventive treatment with ASXSP ameliorates LPS-induced microglia activation in the mPFC. The relative integrated density (RID) of the CD68 positive cells in (**A**) the mPFC area. (**B**) The representative photomicrographs of CD68 staining in the cortex in the different experimental groups. Data are expressed as mean ± SEM (*n* = 10 animals/group). ★, *p* < 0.05 vs. vehicle saline group. @, *p* < 0.05 vehicle LPS group (one-way ANOVA).

Microglia activation is crucially involved in neuroinflammation-induced cognitive impairment. The changes in microglia activation were evaluated using an immunohistochemistry approach in two relevant areas of the CNS involved in learning and memory, the hippocampus (the entire hippocampus and the CA1 and DG areas were analyzed separately) and mPFC (Figure 3 and Figure 4).

Statistical analysis revealed significant differences between the experimental groups in the entire hippocampus (F_(3,33)_ = 6.694, *p* < 0.05). Further, the one-way ANOVA revealed a change between the control vehicle saline and vehicle LPS animals (F_(1,15)_ = 7.605, *p* < 0.05). Changes in the CA1 and CA3 were also observed. The two-way ANOVAs demonstrated a difference between the experimental groups in both regions of the hippocampus (CA1: (F_(3,35)_ = 4.388, *p* < 0.05), CA3: (F_(3,35)_ = 9.442, *p* < 0.05). The posterior one-way ANOVAs revealed a significant difference between the vehicle LPS and ASXSP LPS animals in the area CA3 (F_(1,18)_ = 9.747, *p* < 0.05) and also between the vehicle saline and vehicle LPS rats in both the CA1 (F_(1,17)_ = 4.771, *p* < 0.05) and CA3 (F_(1,17)_ = 10.483, *p* < 0.05) regions.

Analysis of the results obtained in the mPFC show a significant effect of the different treatments (two-way ANOVA: F_(3,31)_ = 7.343, *p* < 0.05). The posterior one-way ANOVAs revealed a difference in microglia activation between the vehicle saline and vehicle LPS (F_(1,15)_ = 7.479, *p* < 0.05) and between the vehicle LPS and ASXSP LPS (F_(1,16)_ = 17.440, *p* < 0.05) experimental groups.

### 3.3. Exposure to LPS and ASXSP Modulates Gut Microbiota Content

**Figure 5:**
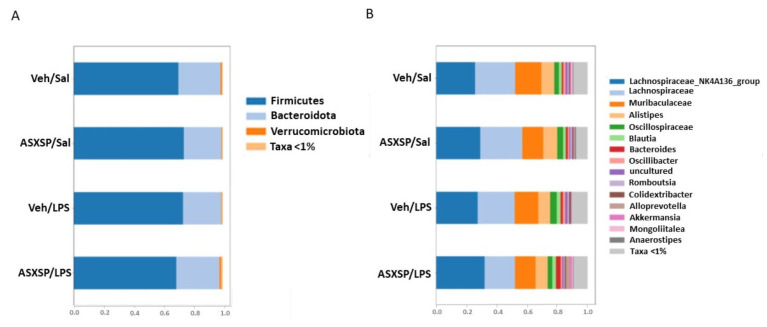
Alterations in gut microbiota content after exposure to LPS and ASXSP. (**A**) Relative proportion of the different bacteria phylum among the experimental groups. (**B**) Relative proportion of the bacteria genus among the experimental groups.

Analysis of the cecum stool samples revealed that the microbiota content was mainly composed of *Firmicutes* and *Bacteroidetes* and a small proportion of *Verrucomicrobia* (Figure 5A). No statistically significant differences were found between the experimental groups at the phylum level. Exploring deeper taxonomic categories, the Lachnospiraceae family was found to be the more abundant among all the experimental groups, followed by the Muribaculaceae and Oscillospiraceae families. In fact, the Oscillospiraceae abundances showed statistically significant differences among the vehicle LPS and ASXSP LPS groups (non-corrected, *p* < 0.03). The Bacteroidaceae family also presented statistically significant differences between the ASXSP LPS and control vehicle saline groups (Table 1). It is important to note the high proportion of the Prevotellaceae family in the ASXSP LPS compared with the other experimental groups, a difference that was mainly due to the *Alloprevotella* genus (Figure 5B). The ASXSP LPS also presented a significantly higher proportion of the Bacteroidaceae family (composed only by the *Bacteroides* genus), and a greater abundance of the Akkermansiaceae family (mainly the *Akkermansia* genus) and Clostridiaceae family, although those differences were not statistically significant. These increments were compensated by a significant decrease in the Oscillospiraceae family and a non-significant decrease in the Lachnospiraceae family. Interestingly, the Cyclobacteriacea family also showed significant differences between the control vehicle saline and vehicle LPS treated rats. The abundance of this microbiota family was significantly reduced in the vehicle LPS rats when compared to the control animals. Of relevance, exposure to ASXSP increased the % in the LPS rats.

Overall, these results show that exposure to LPS led to an increase in microglia activation in the hippocampus and mPFC, and modifications in the gut microbiota. Pretreatment with ASXSP reduced the level of activation of the microglial cells in both areas for rats exposed to LPS. On the other hand, exposure to LPS or ASXSP alone, or in combination, also modified the gut microbiota content. These results suggest that ASXSP beneficial outcomes may involve modulatory effects on microglia activities and gut microbiota.

## 4. Discussion

In the present study we provide insights on the beneficial neuroprotective effects of a treatment with ASXSP, a combination of ASX (administered in a dose range equivalent to human consumption) microencapsulated with spirulina in rats exposed to LPS. ASX was administered to rats at 1.2 mg/kg/day by oral route, a dose selected according to clinical evidence demonstrating that diet supplementation with 12 mg/day of this compound exerted positive effects on different cognitive tasks in humans [31,32,33]. Moreover, this dose was lower than those previously used in other preclinical studies. In this study ASX was administered in combination with spirulina (66 mg/kg/day). Previous studies have reported beneficial effects associated with the consumption of this alga [24,25]. However, to our knowledge, this is the first study trying to elucidate the favorable effects of the consumption of both compounds together. Our results show that the combination of both ASX and spirulina produce important beneficial effects that might involve a synergistic mechanism that potentiates the anti-inflammatory and antioxidant efficiency of these compounds.

Thus, we show that exposure to LPS produced important cognitive dysfunctions and an increase in microglial cells activation in two relevant areas of the CNS involved in learning and memory, the hippocampus and mPFC. Aberrant microglia activation was evaluated as changes in the expression of CD68, a lysosomal protein mainly expressed in activated microglia [38]. Previous studies have reported similar neurophysiological alterations after treatment with LPS, including alterations in microglia cells functionality and a subsequent increase in the synthesis and release of proinflammatory factors and cytokines, such as IL-1β, TNF-α, or BDNF in the CNS [28,29]. These cytokines contribute to neuroinflammation and are directly involved in the cognitive affectations associated with LPS [39].

We also provide insights on the important neuroprotective effects of the preventive treatment with ASXSP, which ameliorated LPS-induced cognitive impairment in the MWM paradigm in rats. These beneficial effects were mediated by the modulation of microglia activities in the hippocampus and mPFC, and possibly by an indirect mechanism that involves modifications in the gut microbiota content. Previous research has reported positive effects from administering ASX or spirulina separately, as they can inhibit aberrant microglia activation. In fact, a recent study described that ASX (at 30 or 50 mg/kg/day p.o.) improved mice cognitive performance in the MWM after exposure to LPS [40]. The authors reported that treatment with ASX could reverse LPS neuroinflammation, amyloidogenesis, and oxidant activity by modulating the activity of the transcription factor “activator of transcription 3” (STAT3) in microglia cells, a mechanism that improves learning and memory processes. On the other hand, spirulina can also decrease LPS-induced microglia dysfunction by regulating the NF-κβ and Nrf2 intracellular pathways [41]. Moreover, previous studies have also demonstrated that phycocyanin, a pigment protein abundant in spirulina, is a selective inhibitor of cyclooxygenase-2 (COX-2), a mechanism by which spirulina can also exert its anti-inflammatory activity [42]. However, to our knowledge, our study is the first to report the beneficial effects from the combination of both compounds. Moreover, our results demonstrate that a low dose of ASX in combination with spirulina produces these important beneficial effects in the subject, suggesting that a synergistic relationship might occur or similar.

On the other hand, we also observed modifications in the composition of the gut microbiota between the experimental groups. Gut microbiota plays an important role in the regulation of the immune system and brain physiology. In fact, gut dysbiosis can alter the functionality of the CNS, affecting learning and memory processes. In this sense, recent studies have shown that the microbiota in Parkinson’s disease (PD) patients is constituted by higher levels of Enterobacteriaceae and decreased levels of *Bacteroidetes* and Prevotellaceae [43]. Moreover, this finding was associated with widespread microglial activation in the basal ganglia and the temporal and frontal cortex in PD patients compared to controls [44]. Our results demonstrate that ASXSP reduced LPS-induced cognitive impairment and neuroinflammation by inhibiting microglial activation. Interestingly, the observed increase in Prevotellaceae content in LPS rats exposed to ASXSP allows us to hypothesize that this change might be within the mechanisms of action of this carotenoid against LPS-induced cognitive dysfunction. Moreover, different bioactive compounds have been shown to exert positive effects on the central nervous system by remodeling the gut microbiota. For instance, quercetin, which is known to improve cognitive behavior in rats [45], was negatively correlated with Oscillospiraceae in healthy, elderly human Japanese subjects [46]. Similarly, ASXSP reduced the Oscillospiraceae abundance when compared to the vehicle LPS group. Finally, the Cyclobacteriaceae content was also modified in the vehicle LPS animals when compared to the control vehicle saline group. Exposure to ASXSP reverted the effect of the exposure to LPS, suggesting a possible involvement in the beneficial effects from the ingestion of the combination of this carotenoid and microalga. Overall, the data implies that the ASXSP protective effect against LPS-induced cognitive deficit may also involve changes in the abundance of several families of gut microbiota. The mechanisms responsible for the beneficial effects of the gut microbiota on CNS functionality have not been fully established, but mounting evidence supports an important role for microbiota-generated short-chain fatty acids [47]. In our experimental conditions, however, no relevant alterations in blood SCFA levels were observed (see Figure S1), suggesting that other mechanisms may be involved. Gut microbiota can also metabolize tryptophan and change the tryptophan availability in the host [48]. Alterations in tryptophan metabolism can also modulate relevant neurobiological functions, including cognition. However, no relevant modifications in the tryptophan content and the related metabolites (5-HT, kynurenine, and 5-HIAA) were observed in the brainstem, hippocampus, or blood in our study (see Figure S2), discarding their possible involvement in the beneficial effects on the performance of the rats in the MWM after ASXSP exposure.

We also evaluated the response of the animals in the Y-Maze and Open Field (see Figure S3) paradigms to evaluate short-term recognition memory and locomotor activity, respectively. No differences were reported in the Y-Maze, suggesting no effects from the exposure to LPS and/or ASXSP modulating short-term recognition memory. The integration and process of short-term recognition memory requires the coordination of different areas of the CNS, including the hippocampus and mPFC [37,49]. However, previous studies have demonstrated that a challenge injection of LPS (1 mg/kg) can increase brain cytokine levels, but produce no significant effects on short-term recognition memory in the Y-maze in mice [50]. In accordance, our results show that, even if microglia activation is still observed both in the hippocampus and mPFC, long after last exposure to LPS (at least until the sacrifice of the rats 16 days later), these alterations seem to play a marginal role in modulating the short-term recognition memory processes of the animals. On the other hand, no differences in locomotor activity were observed between the experimental groups when evaluated in the OF. These data suggest that, in our experimental conditions, LPS or ASXSP alone, or in combination, produce weak activity-wise side-effects. Previous studies have reported hypolocomotion after acute LPS administration in mice. However, tolerance to this behavior develops after repeated exposure to the lipopolysaccharide [51]. We can hypothesize that tolerance to the hypolocomotor effects of LPS may have also developed in our experimental conditions leading to no differences in locomotion between the experimental groups. On the other hand, previous studies have described irrelevant effects from the repeated administration of ASX (in a dose range between 15 and 25 mg/kg/day for 4 weeks) in the locomotor activity of rats [52]. Spirulina also produced no alterations in locomotion when acutely administered in mice (30 mg/kg, p.o.) or chronically (500 mg/kg/day for 30 days) in rats, either. In accordance, no locomotor effects were reported in our study.

Previous studies have reported potent antioxidative and anti-inflammatory effects and the capability to ameliorate cognitive alterations in rodents from both ASX and spirulina [39,40,41]. However, to our knowledge, this is the first investigation demonstrating the important neuroprotective effects from the combination of both bioactive compounds. In the last decades the interest in food supplements has risen, therefore studies performed using a low dose of ASX in combination with spirulina, like this one, could be of relevance.

## 5. Conclusions

To summarize, our results provide insights into the mechanisms of action involved in the neuroprotective effects of ASX microencapsulated with spirulina (ASXSP, referred to as Astagile^TM^). We have observed that treatment with this specific combination of ingredients improves the cognitive performance of the animals after exposure to LPS. ASXSP also decreased LPS-induced neuroinflammation at the level of the hippocampus and mPFC, two brain areas critically involved in learning and memory processing. Moreover, these compounds were able to produce some beneficial prebiotic effects, modifying the gut microbiota content, and decreasing LPS-induced dysbiosis. Overall, our data suggest that microencapsulation of ASX with spirulina may improve ASX antioxidative effects by a mechanism that involves an increase in ASX stability, and/or a synergistic effect from the combination of ASX and phycocyanin and other compounds present in spirulina. However, further studies are required to ascertain the synergistic mechanisms involved in these actions.

## Figures and Tables

**Table 1:** Obtained *p*-values when the microbiota families were compared between the groups. Superscript a indicate statistically significant *p*-values (non-corrected). Relative abundance superscripted with b indicates relevant changes in the relative abundance of a given microbiota family.

	*p Values* (*Non-Corrected*)							*Relative Abundance * (*Mean per Group*)			
	ASXSP/Sal vs. ASXSP/LPS	ASXSP/Sal vs. Veh/LPS	Veh/LPS vs. ASXSP/LPS	Veh/Sal vs. ASXSP/LPS	Veh/Sal vs. ASXSP/Sal	Veh/Sal vs. Veh/LPS		Veh/Sal	Veh/LPS	ASXSP/Sal	ASXSP/LPS
***Prevotellaceae***	0.01 ^a^	0.60	0.02 ^a^	0.13	0.10	0.29		0.06%	0.02% ^b^	0.02% ^b^	2.84% ^b^
***Tannerallaceae***	0.06	0.55	0.26	0.55	0.29	0.41		0.37%	0.55%	0.69%	0.31%
***Akkermansiaceae***	0.13	0.55	0.07	0.60	0.45	0.29		0.81%	0.42%	0.33%	1.10%
***Lachnospiraceae***	0.17	0.26	0.55	1.00	0.33	0.94		57.69%	58.40%	63.02%	56.72%
***Bacteriodaceae***	0.23	0.60	0.29	0.05 ^a^	0.55	0.50		0.95% ^b^	1.35%	1.27%	3.17% ^b^
***Peptostreptococaceae***	0.41	0.94	0.23	0.65	0.88	0.94		0.95%	1.15%	1.29%	0.66%
***Clostridiaceae***	0.41	0.33	1.00	0.71	0.60	0.50		0.18%	0.19%	0.12%	1.02%
***Rikenellaceae***	0.55	0.41	0.94	0.55	0.82	0.71		8.53%	7.77%	9.35%	8.07%
***Muribaculaceae***	0.65	0.41	0.45	0.45	0.36	0.76		17.44%	15.86%	13.69%	13.62%
***Ruminococcaceae***	0.65	0.20	0.29	0.76	0.41	0.33		1.56%	1.75%	1.27%	1.45%
***Cyclobacteriaceae***	0.65	0.06	0.20	0.45	0.65	0.03		1.04% ^b^	0.10% ^b^	0.27%	0.73%
***Oscillospiraceae***	0.76	0.11	0.03 ^a^	0.94	0.71	0.11		6.23%	8.45% ^b^	6.12%	5.81% ^b^

## Supplementary Materials

The following supporting information can be downloaded at: https://www.mdpi.com/article/10.3390/nu15132854/s1, Figure S1: Short Chain Fatty Acids (SCFA) content in feces; Figure S2: Quantification of different tryptophan metabolites in the brainstem, hippocampus, and blood; Figure S3: Locomotor activity measurements evaluated in the Open Field paradigm.
