# Supplementary material for: Preventive Treatment with Astaxanthin Microencapsulated with Spirulina Powder, Administered in a Dose Range Equivalent to Human Consumption, Prevents LPS-Induced Cognitive Impairment in Rats
Source: Nutrients. 2023 Jun 23;15(13):2854. doi: 10.3390/nu15132854 (PMC10343420; doi:10.3390/nu15132854)
Supplement: Supplementary file 1 [file nutrients-15-02854-s001.zip › nutrients-2448688 -Supplementary figure .pdf]

Supplementary figures:

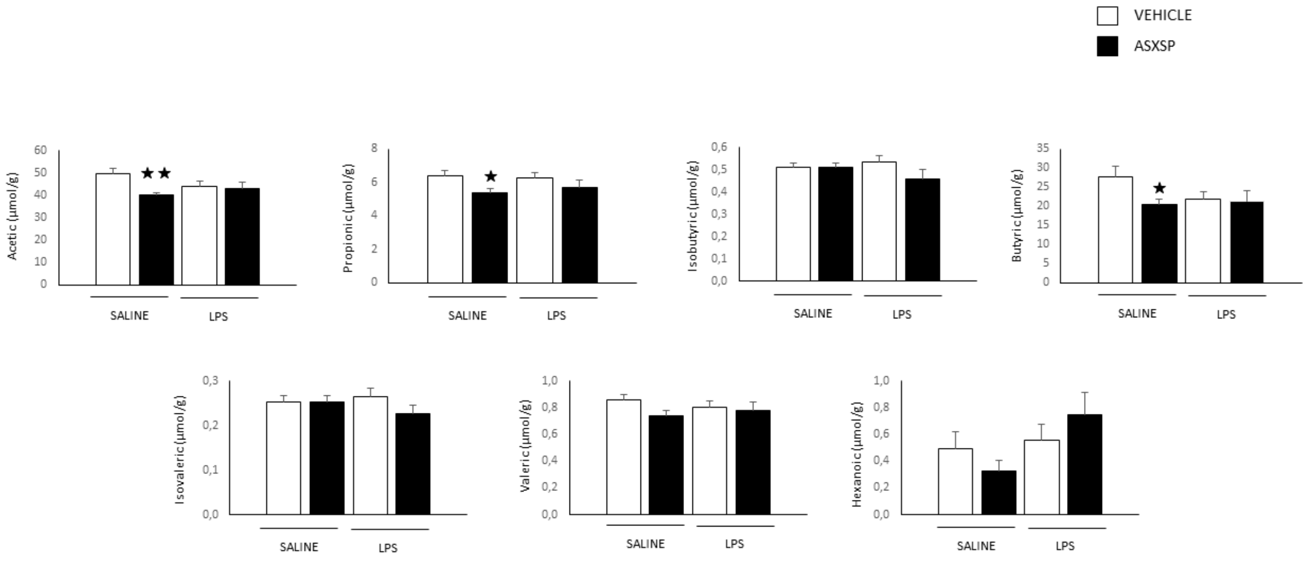

Figure S1. Short chain fatty acids (SCFA) content in feces. Exposure to LPS or ASXSP alone or in combination produced minor changes in SCFA fecal content. Data are presented as the mean ± SEM (n = 10 animals/group). ★, ★★, $p < 0.05$ or $p < 0.01$ vs Vehicle saline group, respectively (one-way ANOVA).

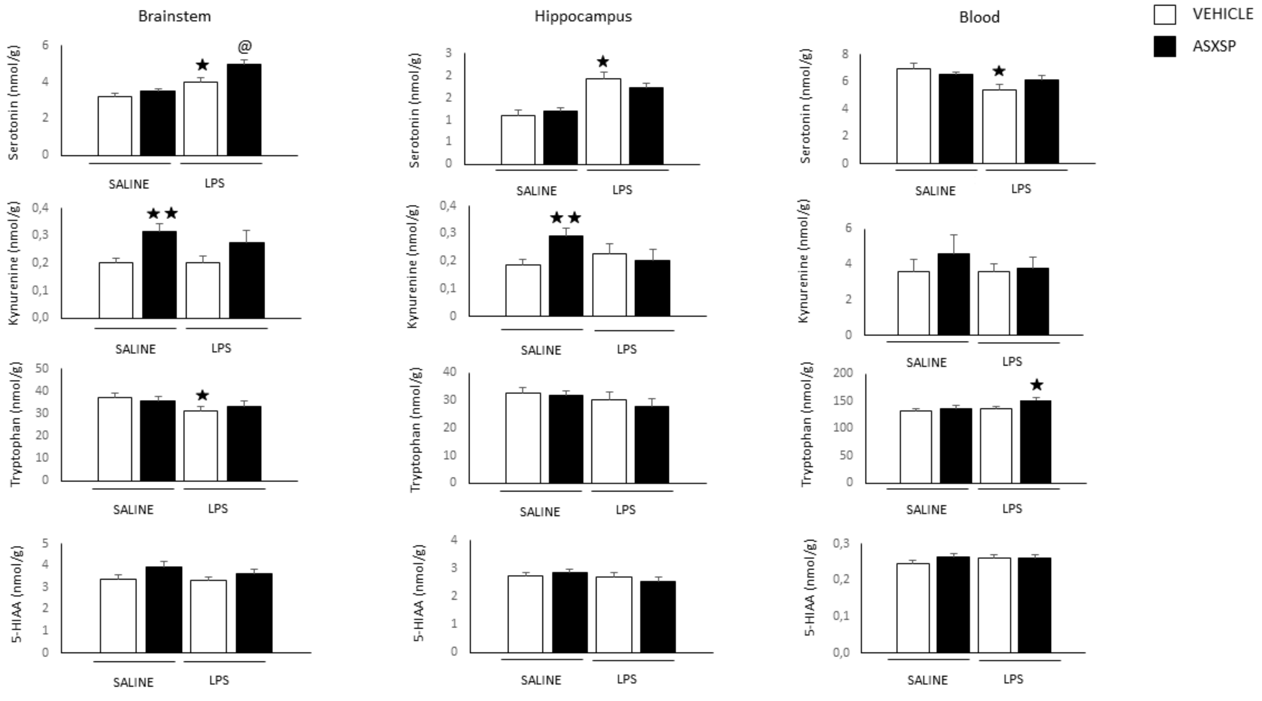

Figure S2. Quantification of different tryptophan metabolites in the brainstem, hippocampus, and blood. Exposure to LPS or ASXSP alone or in combination produced minor changes in tryptophan metabolism in the brainstem, hippocampus and blood in the different experimental groups. Data are presented as the mean ± SEM (n = 10 animals/group). ★, ★★, $p < 0.05$ or $p < 0.01$ vs Vehicle saline group respectively; @, $p < 0.05$ vs Vehicle LPS group (one-way ANOVA).

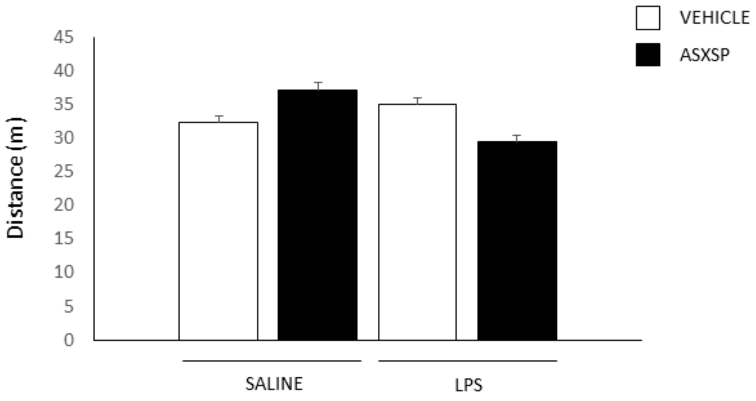

Figure S3. Locomotor activity measurements evaluated in the Open Field paradigm. Exposure to LPS or ASXSP alone or in combination produced no modifications in the locomotor activity in the Open Field in the different experimental groups. Data are presented as the mean ± SEM (n = 10 animals/group).
